# MRI-based patient selection for active surveillance in prostate cancer using U-Found: a generalized deep learning model

**DOI:** 10.1186/s40644-026-00988-z

**Published:** 2026-01-19

**Authors:** Noah C. Lowry, Adrian L. Breto, Veronica Wallaengen, Ahmad Algohary, Nicolas Tapia-Stoll, Sandra M. Gaston, Nachiketh S. Prakash, Pedro F. S. Freitas, Oleksandr N. Kryvenko, Patricia Castillo, Joel Saltz, Tahsin Kurc, Chad R. Ritch, Bruno Nahar, Mark L. Gonzalgo, Dipen J. Parekh, Brandon Mahal, Benjamin O. Spieler, Alan Dal Pra, Matthew C. Abramowitz, Alan Pollack, Sanoj Punnen, Radka Stoyanova

**Affiliations:** 1 Department of Radiation Oncology, University of Miami Miller School of Medicine, 1475 NW 12th St, Miami, FL 33136 USA; 2 Desai Sethi Urology Institute, University of Miami Miller School of Medicine, Miami, FL USA; 3 Sylvester Comprehensive Cancer Center, University of Miami Miller School of Medicine, Miami, FL USA; 4 Department of Pathology and Laboratory Medicine, University of Miami Miller School of Medicine, Miami, FL USA; 5 Department of Radiology, University of Miami Miller School of Medicine, Miami, FL USA; 6 Department of Biomedical Informatics, Stony Brook University, New York, NY USA

**Keywords:** Deep learning, Foundation models, Contrastive learning, Unsupervised deep learning, Prostate cancer, Active Surveillance, Multiparametric (mp)MRI, Diffusion-weighted imaging, Apparent Diffusion Coefficient

## Abstract

**Background:**

Current MRI prostate cancer risk assessment methods focus mainly on detecting tumor lesions, ignoring the prostate gland macro-environment which may also impact disease progression. A generalized deep-learning model for prostate may help capture these gland-level characteristics through deep embeddings which can be used for a variety of downstream tasks. This study aims to assess whether U-Found, a generalized multiparametric (mp)MRI-based model, offers added value in predicting histopathological progression in active surveillance (AS) patients. The prostate macro-environment, captured in U-Found embeddings, is hypnotized to play a significant role in differentiating patients who progress to definitive treatment from those whose tumor is kept at bay.

**Methods:**

U-Found was trained on a dataset comprising over 3000 mpMRIs from in-house and public sources using self-supervised learning. Axial slices were represented in a 128-dimentional space. The physical interpretation of the embeddings was explored by investigating images that are closest to the centroid of embeddings clusters. U-Found was tested on a downstream task: identifying cancer in an independent dataset (publicly available UCLA dataset, *n* = 1,151). To determine the added value of U-Found embeddings to clinical and intratumoral radiomics features, we compared models for predicting histopathological progression in 144 participants of a prospective AS trial. In addition, associations between U-Found embeddings and lesion- and prostate radiomics were investigated.

**Results:**

Our findings suggest that U-Found captures key characteristics of the prostate gland’s macro-environment. U-Found successfully detected cancer in an independent UCLA dataset without being explicitly trained for lesion detection (AUC = 0.79). The prediction model incorporating a combination of clinical variables, mpMRI-derived intratumoral radiomics features and deep embeddings generated by U-Found achieved AUC = 0.86, outperforming models solely based on clinical and/or radiomics features. There were clear associations between U-Found embeddings and radiomics features.

**Conclusions:**

U-Found was designed as a generalized self-supervised foundation model for prostate imaging, enabling the model to learn intrinsic imaging structures. We demonstrate that U-Found embeddings capture key features of the prostate macro-environment, which appear to contribute to disease progression, albeit to a lesser extent than tumor-specific imaging features.

## Background

Multiparametric MRI (mpMRI) of the prostate has improved prostate cancer detection, resulting in the diagnosis of 30% more high-risk cancers than standard template biopsies [1]. The Prostate Imaging-Reporting and Data System (PI-RADS) mpMRI assessment [2], as well as numerous computer-aided diagnosis (CAD) techniques focus mainly on detection and characterization of the tumor lesion. While tumor severity is indeed the main driver of disease progression, the prostate gland condition and macro-environment may also have an impact. Simple reviews of prostate imaging uncover the uniqueness of each exam; prostates are different in terms of size and composition (e.g. percent volume of peripheral vs. transition zone) as well as appearance in terms of image intensities and content (benign prostatic hyperplasia (BPH), hematoma, calcifications, post-interventional changes). An excellent way to capture this diversity is to train large, unsupervised deep learning (DL) models for prostate MRI analysis. Specifically, foundation models are large-scale machine learning models trained on diverse, extensive datasets, designed to serve as the starting point for a wide range of downstream tasks.

The prostate macroenvironment potentially plays a significant role in patients with low-risk PCa who are candidates for active surveillance (AS). We hypothesize that the overall prostate characteristics play a part in differentiating patients who progress to definitive treatment from those where the tumor is kept at bay. AS patients face the risk of cancer progression necessitating treatment and thus require frequent monitoring, including blood draws (to measure Prostate-Specific Antigen (PSA)) as well as imaging and prostate biopsies every 1–2 years [3, 4]. Each biopsy session is associated with substantial patient discomfort and medical risks, such as sepsis and hospitalization, which impacts patients’ willingness to remain on AS. As the percentage of low-risk PCa patients enrolled in AS has more than doubled in the past decade [5], new diagnostic tools are needed to safely reduce unnecessary repeat prostate biopsies by identifying patients at risk of aggressive disease in order to provide timely treatment.

In this study, a novel generalized model (U-Found) for capturing prostate gland characteristics is presented. U-Found represents each axial mpMRI-image in 128-dimentional space. The resulting 128-dimensional vectors, called embeddings, enable clustering of the images by their similarities and we investigated the patterns “seen” by the network. Appreciating that the explainability of DL models often presents a barrier for clinical application, we further studied the correlation of the embeddings with prostate and lesion radiomics features. We investigated the power of the model to identify cancer without being explicitly trained for detecting lesions in an independent dataset. Finally, we present an approach to identify AS patients at risk of progression based on the integration of clinical variables and intratumoral radiomics features with DL embeddings from U-Found. Imaging and clinical data from patients enrolled in the MRI-Guided Active Selection for Treatment of Prostate Cancer (MAST) trial were utilized to develop the risk assessment models.

## Methods

### U-Found: training dataset and conceptual overview

Prostate mpMRI from the Prostate Imaging: Cancer AI (PI-CAI) challenge [6] and from clinical trials conducted at the University of Miami (UM) were used to train the U-Found encoder model. The UM collections contained mpMRI exams from the Departments of Radiation Oncology and Urology, reviewed under an IRB-approved protocol for retrospective review of prostate imaging (Protocol #20090554). The model was trained on the Apparent Diffusion Coefficient (ADC) exams from 3,244 patients: 1,500 cases provided through the PI-CAI Public Training and Development Dataset and 1,744 UM cases. The MRI scanners used to acquire the images are summarized in Table 1.

**Table 1:** A summary of the different magnets, manufacturers, and field strengths used to acquire multiparametric MRI imaging for the PI-CAI grand challenge’s public dataset and the University of Miami (UM) collection

Magnet	Field Strength (T)	PI-CAI (*n*)	UM (*n*)
GE Discovery MR750	3		601
GE Signa HDxt	1.5		3
GE Signa Pioneer	3		8
Philips Achieva	1.5/3	52	1
Phillips Ingenia	1.5/3	227	2
Siemens Aera	1.5	17	1
Siemens Avanto	1.5	13	5
Siemens Espree	1.5		1
Siemens Essenza	1.5		1
Siemens Lumina	3		1
Siemens Sola	1.5		6
Siemens Vida	3		121
Siemens Prisma	3	89	1
Siemens Skyra	3	1034	679
Siemens Sonata	1.5		11
Siemens Symphony	1.5		10
Siemens TrioTim	3	68	256
Siemens Verio	3		36
**Total**:		1500	1744

**Fig. 1:**
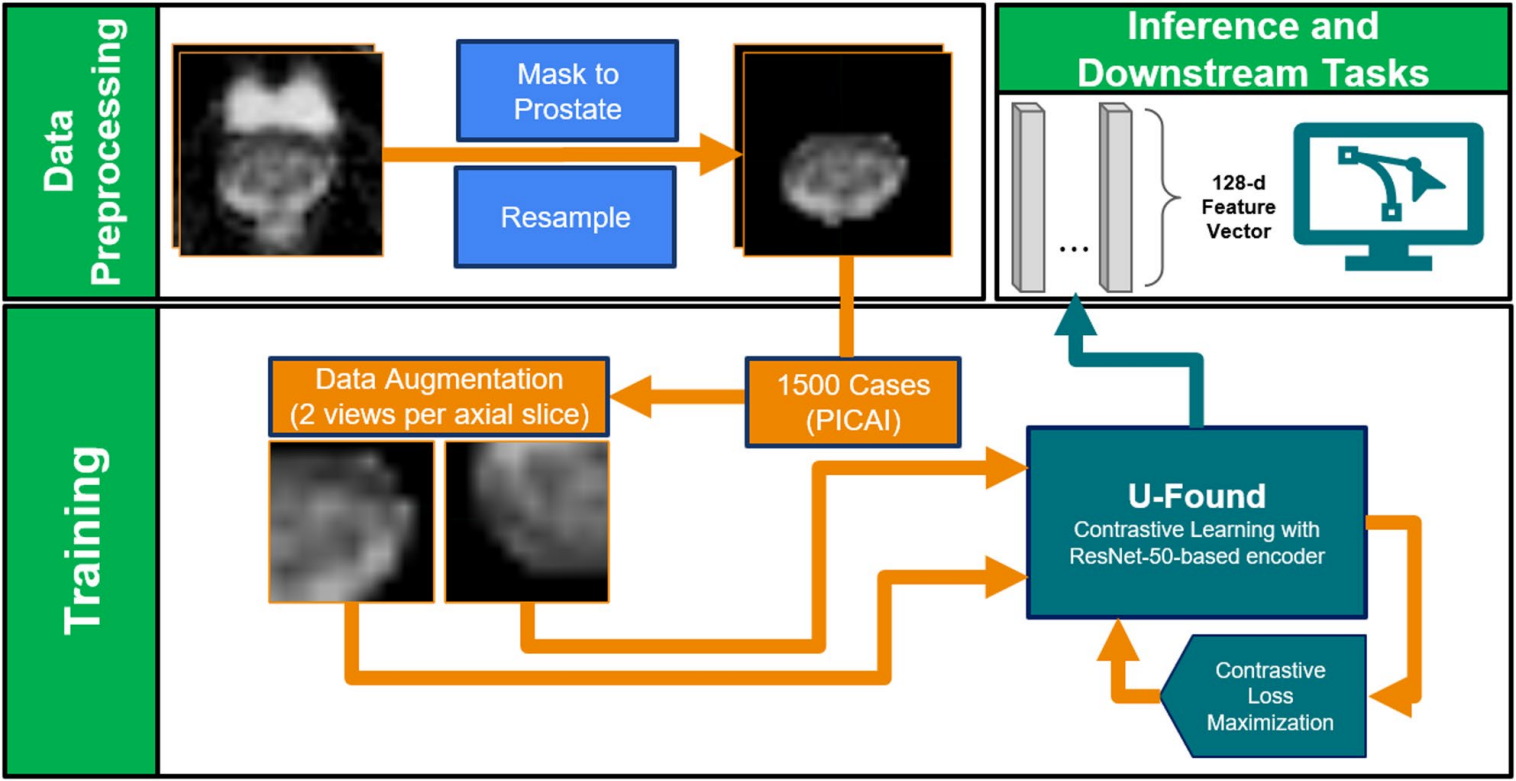
Overview of U-Found training. Axial Apparent Diffusion Coefficient (ADC) slices with visible prostate are masked to the prostate contour and resampled. Each axial slice received different data augmentations and was provided to the network as a positive class (minimizing contrastive loss). The output of the trained network is a 128-dimensional deep feature vector per-image that can be used in further downstream tasks

A high-level overview of U-Found is shown in Fig. 1. U-Found implements contrastive learning, an unsupervised DL method which aims to distinguish between similar and dissimilar data points, using the SimCLR implementation, trained via instance discrimination [7]. SimCLR creates abstract lower-dimensional representations of images with a convolutional neural network called an *encoder.* In the encoder training stage, a pair of randomly subsampled regions of the image are *augmented* by random, differing transformations (cropping, horizontal flipping, etc.) generated from each image in the dataset.

### U-Found: image pre-processing and contrastive learning

The 2D axial ADC images (total of 98,745 images) contained both visible portions of the prostate and regions superior and inferior of the prostate. To prevent the unsupervised learning from identifying and promoting irrelevant extraprostatic features in the final embeddings, slices not containing the prostate were removed from the training dataset. In addition, the images were masked to the prostate using the AI-derived contours provided for the PI-CAI dataset [8] and readily available manual contours for the UM dataset. Additional common preprocessing steps for deep learning on medical images were implemented: percentile-based rescaling of pixel intensities (2nd to 98th percentiles), centering of the images around the prostate centroid, and uniform cropping to 64 × 64 pixels to homogenize the inputs to the network. All preprocessing steps were carried out using the Python MONAI library [9]. The final dataset used for training was composed of 48,938 slices of different resolutions.

For SimCLR a contrastive loss function was constructed, taking on lower values when the cosine similarity between representations of the same images is high and higher when the cosine similarity between representations of different images is low. Contrastive loss was minimized with gradient descent-based methods. The goal of the training was to minimize contrastive loss between view-pairs.

SimCLR was originally proposed as a method applied to standard image formats in computer vision. We made several modifications to account for the differences between standard images and MRI data:

1. SimCLR includes random color jittering and random grayscale conversion as part of its transformation pipeline. We removed these transformations due to the inherent grayscale nature of MRI intensities. We also removed Gaussian blurring from the pipeline as we found blurring to disrupt the ability of the model to learn features from images of a small size. Thus, our transformation pipeline consisted of random cropping, random translation, and random reflection.
2. SimCLR was trained on 224 × 224 pixel images to produce 2048-dimensional vector representations. To account for the relatively reduced information contained in our 64 × 64 grayscale images, we scaled the output size of the encoder down to 128 dimensions.

The encoder network architecture has a ResNet-50 instance and was initialized with ImageNet-1 K-pretrained weights. Non-random weight initialization is beneficial for increased encoder reproducibility and supported by previous studies that suggest good performance of natural-to-medical image domain transfer [10]. The encoder was trained in batches of 128 images for 600 epochs and optimized with the AdamW algorithm and a cosine learning rate schedule. Hyperparameter tuning via the Optuna framework yielded an optimal learning rate of 3.6 × 10^− 4^, weight decay of 1.3 × 10^− 9^, and loss function temperature parameter of 0.07 (from the common range [0.07, 0.2, 0.5]) [11]. All network training was implemented with PyTorch 2.2.1 [12] and MONAI 1.3.0 for Python on a system with a 24 GB-VRAM NVIDIA RTX 3090 GPU and 64 GB of RAM. Post-training, the 128 dimensional “embedding space” was explored with the Uniform Mapping and Projection Algorithm (UMAP) [13] and k-means clustering.

### U-Found downstream task: identification of cancer in independent dataset

We investigated the power of U-Found embeddings to identify cancer in the Prostate-MRI-US-Biopsy dataset [14] (UCLA dataset, *n* = 1,151). Complete prostate and lesion DICOM contours were available for 807 exams, which were preprocessed in the same manner as the PI-CAI and UM datasets. The UCLA dataset comprised 12,460 uniformly resampled 2D axial slices of 64 × 64 pixels.

### Miami MAST trial

“MRI-Guided Biopsy Selection of PCa Patients for Active Surveillance versus Treatment: The Miami MAST Trial” (ClinicalTrials.gov: NCT02242773) is a prospective, single-center, single-arm, non-therapeutic interventional trial for men undergoing AS for PCa. Patients aged 35–85 years with biopsy-confirmed PCa within 18 months and PSA ≤ 20 ng/mL within 3 months were enrolled between 2014 and 2020. To meet inclusion criteria, diagnostic biopsies must have had a minimum of 8 cores with 4 or fewer positive cores, and 2 or fewer cores of Grade Group (GG) 2 cancer and be centrally reviewed by a genitourinary (GU) pathologist (ONK). Exclusion criteria included any core with GG3 or higher cancer, extraprostatic extension on digital rectal exam, prior pelvic radiation, bilateral hip replacement, and concurrent malignancy.

The study protocol entailed an mpMRI and MRI-Ultrasound guided (MRI-US) biopsy (Confirmatory) within 18 months of the diagnostic biopsy, followed by surveillance biopsies at 12, 24, and 36 months, or until histopathologic progression occurred. The confirmatory MRI-US session, performed immediately after enrollment, defines the start of the trial timeline and serves as the *baseline* for all subsequent progression assessments. The initial diagnostic biopsy is used exclusively for eligibility determination and is not incorporated quantitatively into progression analyses. This is because diagnostic biopsies are typically performed outside the trial, often without MRI- US guidance, and under variable technical and interpretive conditions across institutions and practice settings.

Histopathological progression is defined as one or more of the following: *(i)* more than 4 cores with any grade cancer, *(ii)* more than 2 cores with GG2 cancer, *(iii)* any single core with GG3 or higher cancer, *(iv)* a GG1 at diagnosis upgraded to GG2 or higher.

Two groups were identified among the MAST participants:

i. **Rapid progressors**: Participants who progressed within 12 months of their confirmatory, baseline visit.
ii. **Slow progressors**: Participants who progressed at 24 or 36 months of their baseline visit or who completed the full trial without signs of histopathological progression.

MRI sequences and sequence parameters were consistent with the recommendations for PI-RADS v2 [15]. The exams consisted of axial T2-weighted MRI of the pelvis, Diffusion-Weighted Imaging (DWI) with the generation of ADC maps and Dynamic Contrast Enhanced (DCE)-MRI. Suspicious-for-cancer regions were outlined and targeted in Dynacad (InVivo, Gainesville, FL) by a radiologist with more than fifteen years of experience in GU malignancies (PC) using PI-RADSv2. MRI-US biopsies were carried out using UroNav (InVivo, Gainesville, FL). MpMRI targeted (2 cores/lesion) and standard template (systematic) biopsies were collected from each patient. Clinical, imaging and histopathology data was stored in a dedicated RedCap (Qualcomm, San Diego, CA) database.

### Feature extraction and AS progression model

The details of the radiomics extraction pipeline are given elsewhere [16]. Briefly, 11 features (mean, min, max, standard deviation, skewness, kurtosis, 10th /25th /50th /75th /90th percentiles) were extracted from: ADC, T2-weighted, high B-value images from DWI and early enhancing DCE. In addition, volumes of the prostate, the largest lesion (assumed to correspond to the index lesion), and all identified lesions combined, as well as the number of identified lesions were included as features, resulting in a set of 48 total features. Two sets of radiomics features were extracted: from lesion(s) and the entire prostate gland.

The U-Found neural network weights were used to generate the 128-dimensional deep embeddings from the ADC images of the MAST patients. Max pooling was applied to merge features across ADC slices to obtain a single feature vector for each patient.

Feature selection was used to identify the most relevant features with respect to histopathological progression of PCa and applied separately to the lesion radiomics features and the U-Found deep embeddings. The Minimum Redundancy-Maximum Relevance (mRMR) [17] technique was used to select a set of 12 top-ranked features and Exhaustive Feature Search was subsequently utilized to select 3–12 of these features for a logistic regression classifier predicting time to progression based on 5-fold cross-validation. The criterion for the final feature count selection was highest cross-validation AUC.

## Results

### U-Found: clustering and patterns in embedding space

**Fig. 2:**
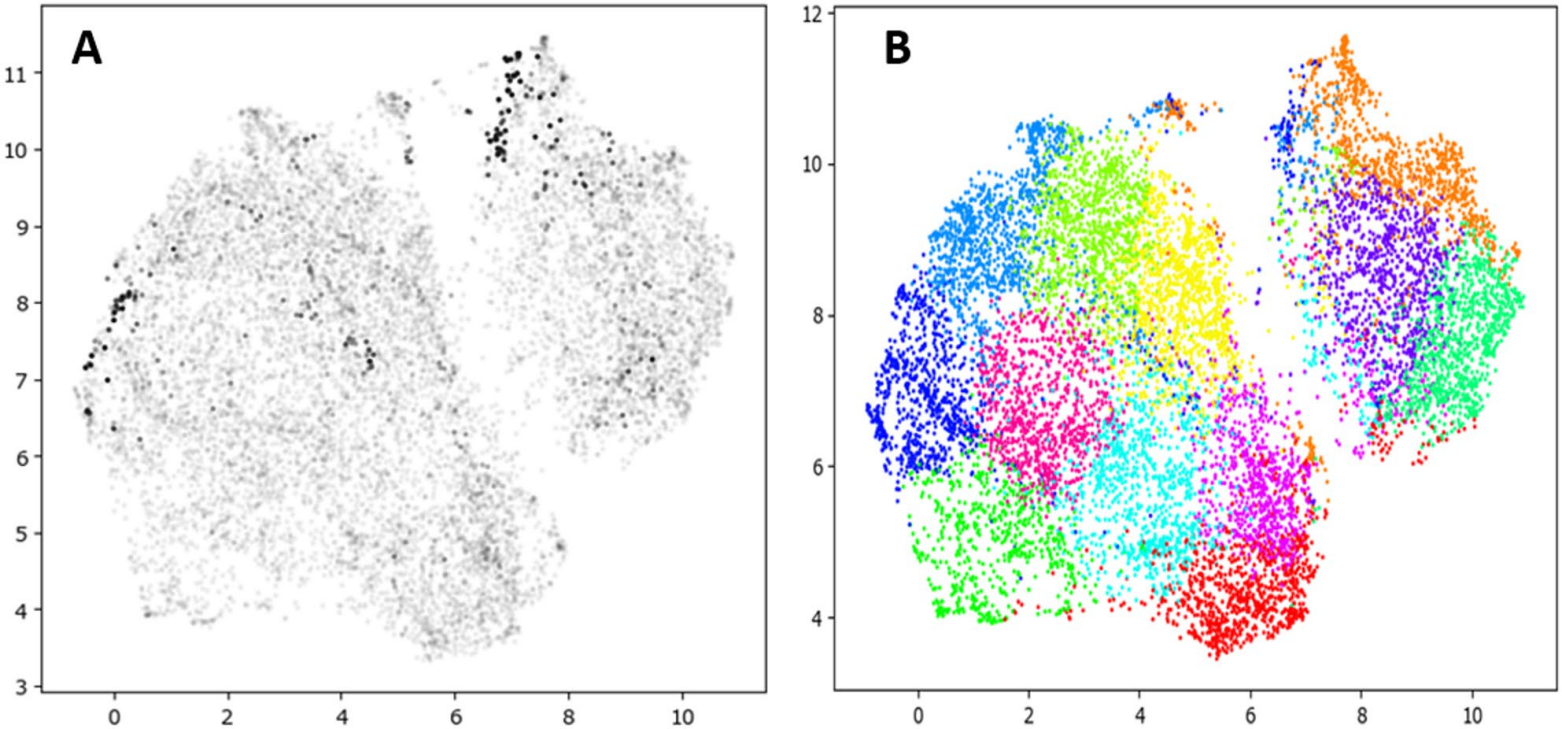
UMAP projection of the 128-dimensional embedding space from the Apparent Diffusion Coefficient (ADC) images in PI-CAI. (A) The grayscale of each point corresponds to the amount of visible tumor lesion on each axial slice; (B) The UMAP divided into 12 clusters via k-means clustering

**Fig. 3:**
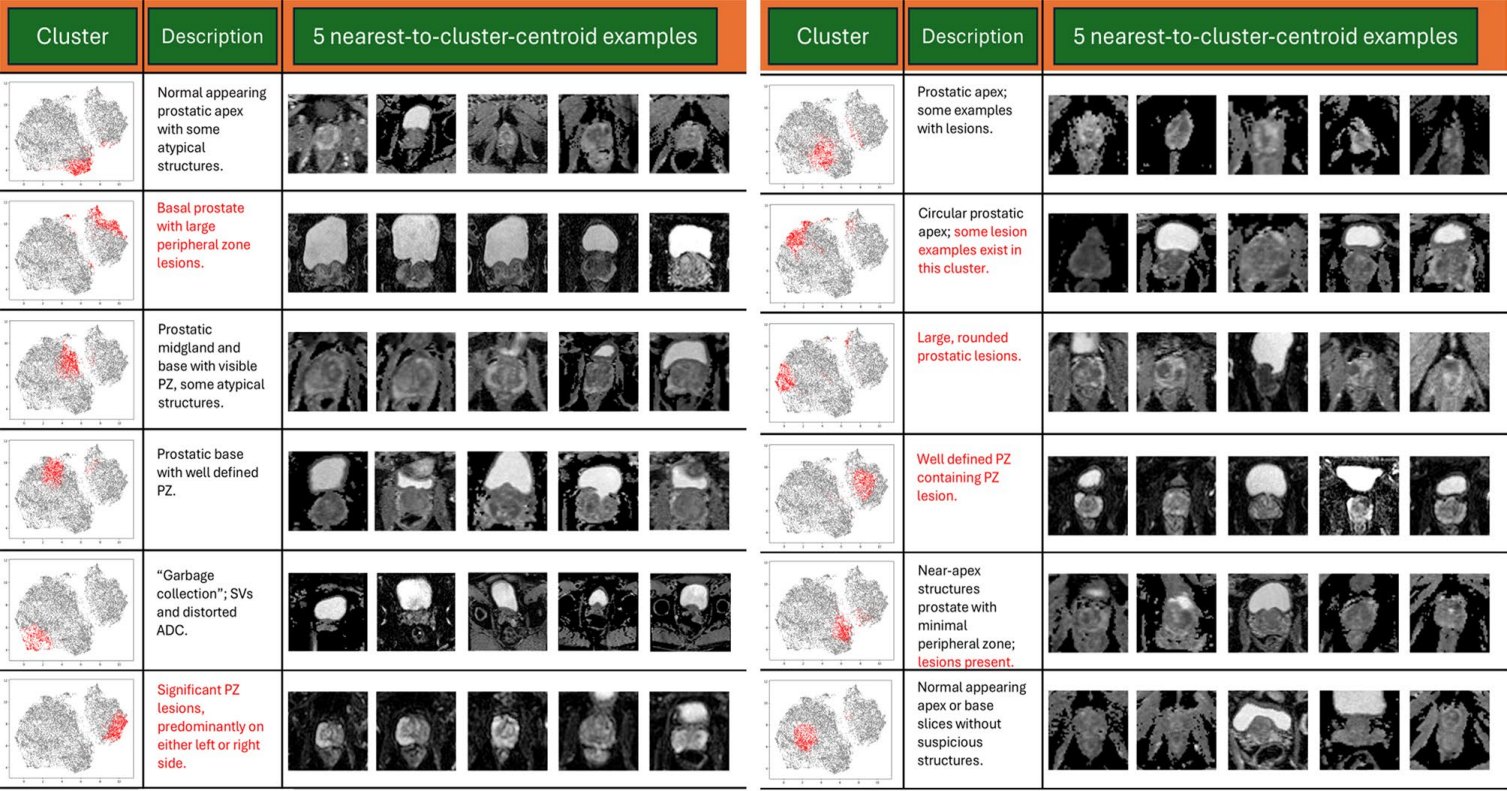
Deep feature interpretability: A sample of 5 images, closest to the centroids of the 12 clusters (Fig. 2B), along with a qualitative description. Cluster members are largely grouped by image characteristics and location within the prostate. Lesions are predominantly found in the smaller “northeast” cluster. Aberrant examples of noisy ADC were also placed into a cluster highly separated from other data (Cluster # 5). Of note, while prostate-masked images were used for training, the entire images are displayed for greater context. Abbreviations: ADC = Apparent Diffusion Coefficient, SVs = seminal vesicles, PZ = peripheral zone

The embeddings from the PI-CAI axial slices (*n* = 22,290) are presented in a 2D plot using the UMAP algorithm (Fig. 2A). The scatter plot was coded in grayscale by the amount of tumor-labeled pixels per slice. While tumor labeling was not used in the training, notably the smaller, north-east cluster contains a denser concentration of tumor lesion slices. To further examine the content of the embeddings, a k-means clustering algorithm was run on the deep feature vectors of the embedding space. Twelve clusters were generated, visualized in Fig. 2B. The contents of the clusters are examined in Fig. 3, visualizing the five closest unique examples (no images from the same patient) to the centroid of each cluster. The smaller cluster is composed entirely of lesion-containing and non-normal appearing axial slices, while the larger cluster is comprised of mostly normal appearing tissue except for one cluster on the opposite side of the embedding space. Notably, aberrant or noise-distorted ADC slices were grouped into their own “garbage collection” cluster (#5) which indicates that SimCLR was able to discern its own filtering criteria against the rest of the dataset as part of its unsupervised learning regime.

### U-Found downstream task: identification of cancer in UCLA dataset

**Fig. 4:**
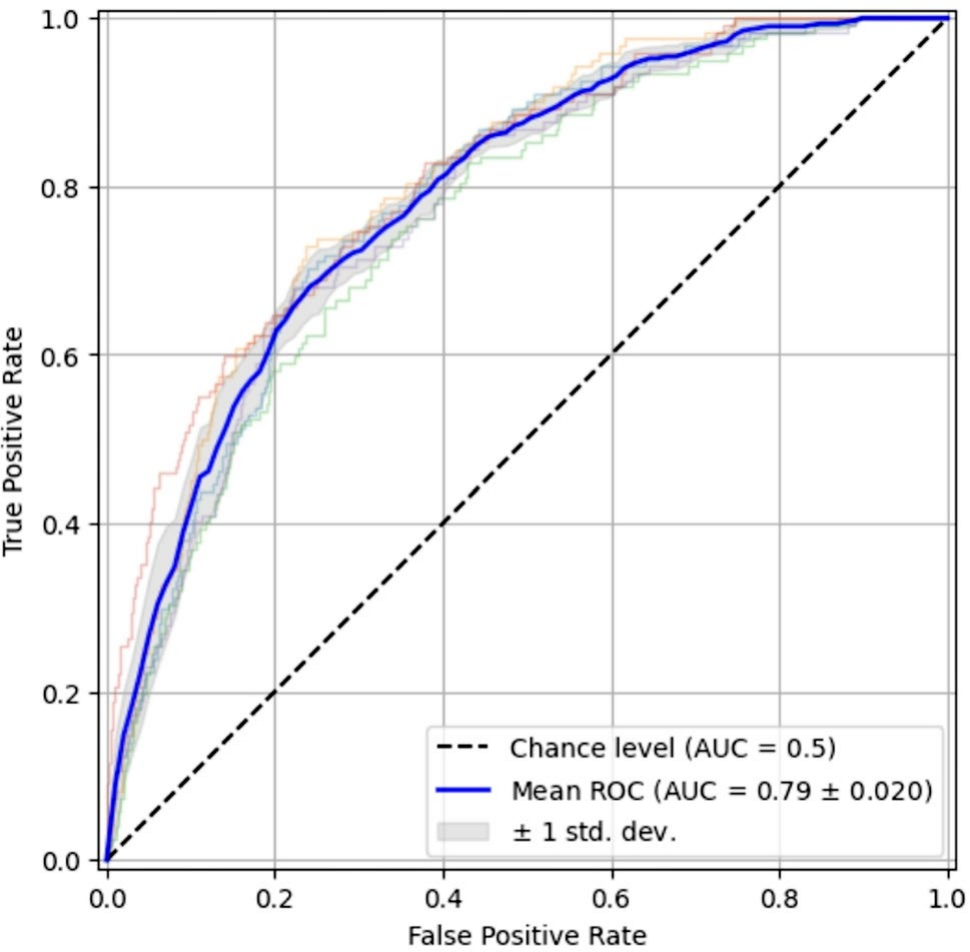
ROC curve from cancer prediction model. Classification (AUC = 0.79) of each axial slice based on cancer vs. no cancer (UCLA dataset). Abbreviations: ROC = receiver operating curve; AUC = area under ROC

Using the lesion segmentation, axial slices were labelled as “cancer” or “no cancer”, based on a 61 pixels threshold. The threshold was determined via histogram analysis of the number of pixels in the lesions. A logistic regression model was built to predict cancer, using mRMR [17] for variable selection. The classifier was trained on the 11 most significant deep features and resulted in AUC = 0.79 using 5-fold cross-validation (Fig. 4**)**.

### Miami MAST trial: AS progression model

One hundred and forty-four participants from the MAST trial had sufficient information to be labeled as *rapid* (*n* = 71) or *slow progressors* (*n* = 73). The patient characteristics are listed in Table 2. In the analyzed cohort, the median time interval between diagnostic and confirmatory biopsy was 8.3 months (IQR: 6.6–11.6; range: 1.4–17.7 months). The relatively high number of progressors at baseline indicates the upgrade detected on the confirmatory MRI-US biopsy at enrollment, reflecting disease that was likely under-sampled at the initial diagnostic biopsy or progressed during the relatively long period between diagnostic and confirmatory biopsy.

A total of 12 features were selected: 4 radiomics (prostate volume, high b-value skewness, ADC 25th percentile, DCE standard deviation), 5 deep embeddings and 3 clinical variables: age, PSA level and highest PI-RADSv2.1 assessment category. Six logistic regression models were trained to distinguish between the two groups, incorporating clinical variables, mpMRI-derived intratumoral radiomics features and deep feature vector embeddings generated by U-Found separately and in combination.

**Table 2:** MAST patient characteristics at baseline visit. *P*-values comparing rapid and slow progressors computed using *t*-test statistics for continuous variables and chi-square test statistics for categorical variables. Percentage of total for each group specified in parentheses for categorical variables

Variable	Total (*N*=144)	Rapid progressors (*n*=71)	Slow progressors (*n*=73)	*P*-value
**Age**,** years**,** median [IQR**,** SD]**	63 [57-70, 8.5]	65 [59-70.5, 8.1]	61 [55-67, 8.3]	0.002
**PSA**,** ng/mL**, **median [IQR**,** SD]**	5.0 [3.7-6.9, 3.6]	5.3 [4.3-8.2, 3.8]	4.5 [3.2-6.3, 3.4]	0.02
**PI-RADSv2.1**				< 0.001
Negative (< 3)	28 (19.5)*	7 (9.8)	21 (28.8)	
3	39 (27.1)	11 (15.5)	28 (38.3)	
4	64 (44.4)	43 (60.6)	21 (28.8)	
5	13 (9)	10 (14.1)	3 (4.1)	
**Grade Group**				< 0.001
Benign	43 (29.9)	7 (9.9)	36 (49.3)	
1	65 (45.1)	29 (40.8)	36 (49.3)	
2	17 (11.8)	16 (22.5)	1 (1.4)	
3	9 (6.3)	9 (12.7)		
4-5	10 (6.9)	10 (14.1)		
**Time of histopathological progression**				< 0.001
Baseline (0 months)	53 (36.8)	53 (74.6)		
1^st^ surveillance (12 months)	18 (12.5)	18 (25.4)		
2^nd^ surveillance (24 months)	5 (3.5)		5 (6.9)	
3^rd^ surveillance (36 months)	2 (1.4)		2 (2.7)	
Completed study w/o progression	66 (45.8)		66 (90.4)	

Abbreviations: IQR = interquartile range, SD = standard deviation

*Benign biopsy results reflect known sampling limitations and do not contradict prior biopsy-confirmed prostate cancer at enrollment

**Fig. 5:**
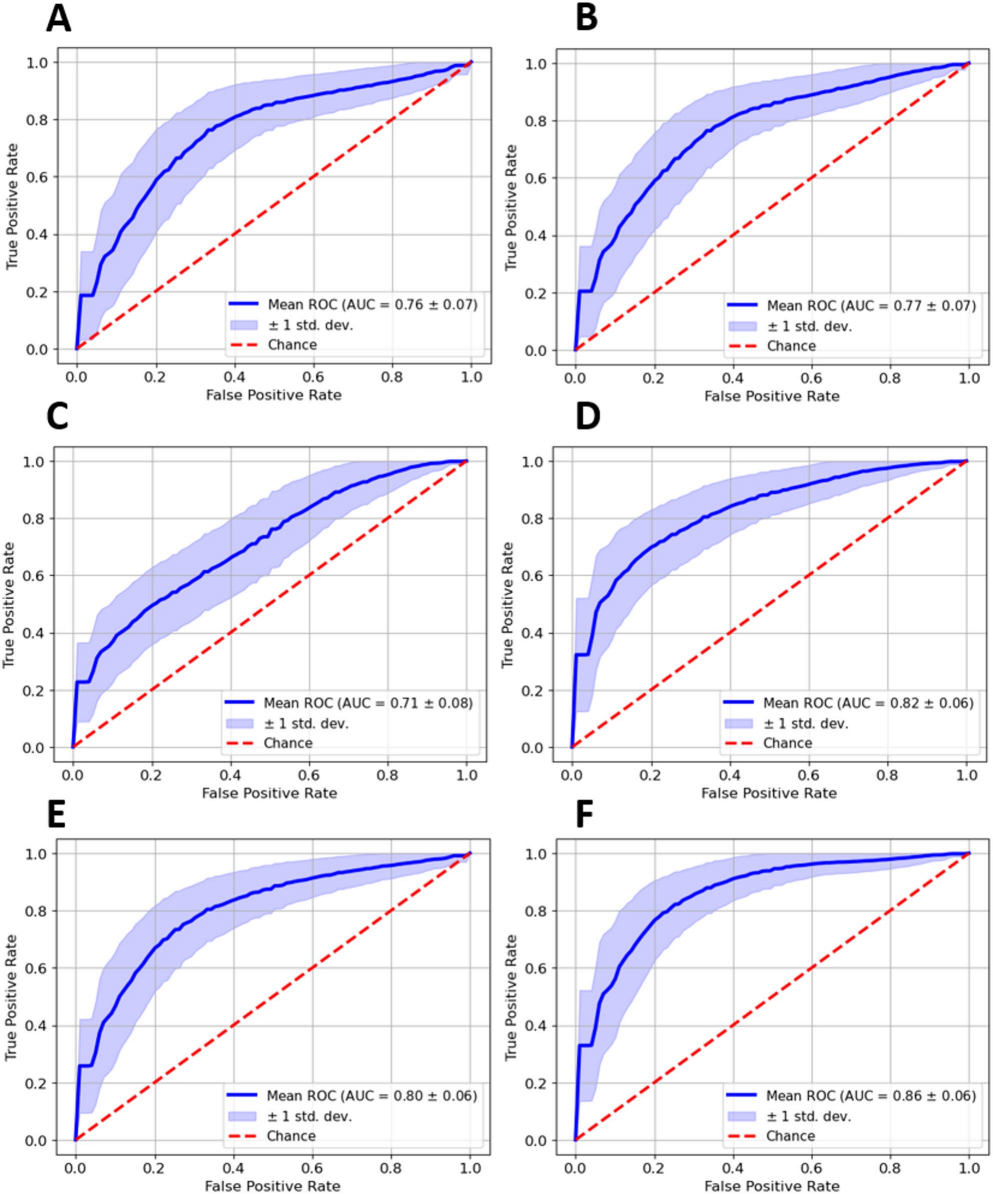
ROC curves and AUCs from progression models. **(A)** Clinical variables; **(B)** Radiomic features; **(C)** U-Found deep features; **(D)** Clinical plus radiomics; **(E)** Clinical plus deep features; **(F)** All combined. Abbreviations: ROC = receiver operating curve; AUC = area under ROC

The AUCs of the logistic regression models are presented in Fig. 5. When trained on the feature sets alone, the mean AUCs were: (**A**) 0.76 ± 0.07 for clinical variables, (**B**) 0.77 ± 0.07 for radiomics features and (**C**) 0.71 ± 0.08 for U-Found embeddings. When the feature sets were combined, the AUCs increased to: (**D**) 0.82 ± 0.06 for clinical + radiomics features and (**E**) 0.8 ± 0.06 for clinical variables + U-Found embeddings. Finally, an AUC of 0.86 ± 0.06 was obtained when all feature sets were combined (**F**). The difference with the next closest AUC (0.82, clinical variables and radiomics features (**D**)) was marked but not statistically significant, most likely due to the relatively small sample size.

**Fig. 6:**
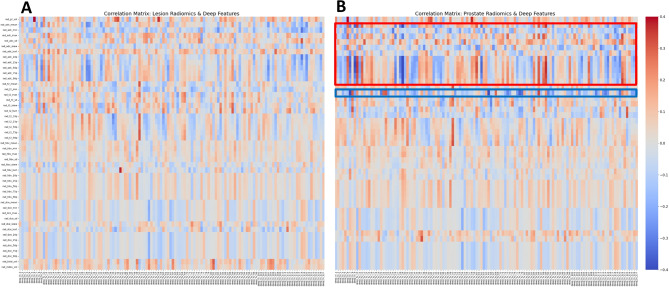
Correlations between DL embeddings (x-axis) and radiomics features (y-axis), displayed as a heatmap. **(A)** U-Found embeddings and radiomics features from cancer lesions; and **(B)** U-Found embeddings and radiomics features from the entire prostate. Red bar indicates correlation with prostate ADC radiomics features; blue bar - T2-weighted minimum feature

The correlation matrices between DL embeddings and (*i*) lesion; and (*ii*) prostate radiomics were computed in order to study the relationships between radiomics and U-Found embeddings (Fig. 6). It is evident that the embeddings show a stronger correlation with prostate-, rather than lesion radiomics features, particularly with the ADC radiomics (indicated by the red bar). Strong correlations were also observed with the T2-weighted minimum feature (blue bar), which may be related to tumor or transition zone signals.

## Discussion

Foundation models are large-scale machine learning models trained on diverse, extensive datasets to perform tasks such as disease detection and image segmentation [18]. Existing foundation models generate embeddings as part of their processing pipeline, but do not consider them an output in of themselves. This is typical of foundation models which specialize in multimodal data, requiring the transformation of imaging features into tokens for large-language model outputs, contours for image segmentation, or to generate multiple predictions from a single set of image patches [19–21].

To the best of our knowledge, U-Found is a unique model, designed as a generalized, self-supervised foundation model for prostate imaging. U-Found was trained using contrastive learning without any outcome, class, or lesion annotations. This training strategy enables the model to learn intrinsic imaging structures and indeed, we demonstrate that U-Found embeddings capture key features of the prostate macro-environment. In contrast, other foundation models are typically trained using supervised or weakly supervised objectives and optimized for a single downstream task, most commonly lesion detection or segmentation [22]. While these approaches demonstrate strong performance for their intended tasks, their learned representations are inherently shaped by task-specific labels. Meanwhile, U-Found can be flexibly applied across multiple downstream tasks, including progression prediction, without retraining or architectural modification.

We chose to generate 128 deep imaging features from our training dataset, selected as a compromise between preserving informative feature heterogeneity and minimizing noise amplification. Higher-dimensional representations risk encoding scanner-specific variability with limited biological relevance, whereas overly compact embeddings may wash out clinically meaningful signals.

Here, we examined the contribution of U-Found embeddings to a contemporary clinical question for the overall selection of patients suitable for AS. The comparatively lower standalone performance of U-Found is likely attributable to its emphasis on global prostate characteristics rather than lesion-specific tumor features. While gland-level representations may capture aspects of the prostate macro-environment that influence disease biology, progression to definitive treatment in active surveillance is more directly driven by lesion-level aggressiveness and clinical factors. Nevertheless, the improvement observed when U-Found features were integrated with radiomic and clinical variables suggests that deep, gland-level embeddings provide complementary prognostic information rather than redundant or competing signals. These results are promising for the development of a model with potential to improve risk stratification in AS patient selection.

Further, we explicitly demonstrated that the deep embeddings are associated with prostate anatomy patterns. We also analyzed the relationship between U-Found embeddings and radiomics features. We observed that the associations were more pronounced when radiomics were extracted from the entire prostate rather than from lesion-specific regions. This suggests that the embeddings primarily encode intrinsic imaging characteristics of the prostate macro-environment, such as global diffusion and tissue heterogeneity, rather than focusing exclusively on localized lesion properties. While exploratory in nature, this analysis provides insight into the biological grounding of the learned representations and supports the hypothesis that U-Found captures meaningful prostate-level imaging features. To our knowledge, this is the first study to use such an embedding-radiomics association framework to investigate the internal representations of a foundation model in prostate MRI.

This study has limitations. Particularly, only one of the mpMRI sequences was used for U-Found training. The ADC was selected as the measurement reflects tissue diffusion properties, measured in direct physical units, unlike the other sequences where the intensity units are arbitrary. In addition, ADC has high diagnostic signal as reflected in the PI-RADS recommendations. However, ADC values can still vary due to scanner-specific factors, signal-to-noise ratio, and fitting methodology. In this study, we applied min-max rescaling of ADC values to the 0–1 interval as part of the deep learning preprocessing pipeline. Because ADC values fall within a defined numerical range (0-3000 × 10⁻⁶ mm²/s), this rescaling preserves relative contrast and does not distort the diffusion-related information. However, this approach is not directly transferable to non-quantitative MRI sequences (e.g., T2-weighted, DWI, or DCE), where intensity ranges vary widely across scanners and acquisition parameters. For these sequences, simple rescaling may lead to loss of biologically relevant contrast. Future work should therefore evaluate sequence-specific normalization strategies and assess their impact on model robustness and generalizability. Further, U-Found was trained using two datasets in which prostate contours were generated using different strategies (AI-derived and manual segmentation). While prior studies - including work from our group - have demonstrated high fidelity of AI-based prostate segmentation relative to expert manual contours (Dice similarity coefficients approaching 0.9), differences in segmentation methodology and preprocessing across datasets may introduce subtle sources of bias.

## Conclusions

U-Found was designed as a generalized, self-supervised foundation model for prostate imaging. U-found was trained using contrastive learning without any outcome, class, or lesion annotations. This training strategy enabled the model to learn intrinsic imaging structures and indeed, we demonstrate that U-Found embeddings capture key features of the prostate macro-environment which contribute to lesion identification and disease progression.

## Data Availability

The datasets generated and/or analyzed during the current study are available from the corresponding author on reasonable request.

## Abbreviations

ADC: Apparent Diffusion Coefficient
AS: Active Surveillance
CAD: Computer-Aided Diagnosis
DCE: Dynamic Contrast-Enhancing
DL: Deep Learning
DWI: Diffusion-Weighted Imaging
GG: Grade Group
GU: Genitourinary
MAST: MRI-Guided Biopsy Selection of PCa Patients for Active Surveillance versus Treatment: The Miami MAST Trial
mp: Multiparametric
MRI-US: MRI-Ultrasound
mRMR: Minimum-Redundancy-Maximum-Relevance
PCa: Prostate Cancer
PI-RADS: Prostate Imaging Reporting & Data System
PSA: Prostate-Specific Antigen
UM: University of Miami
UMAP: Uniform Manifold Approximation and Projection
